# Neurobiological effects and mechanisms of magnetic fields: a review from 2000 to 2023

**DOI:** 10.1186/s12889-024-18987-9

**Published:** 2024-11-08

**Authors:** Xuejia Wang, Yumeng Ye, Hongyan Zuo, Yang Li

**Affiliations:** 1 Department of Experimental Pathology, Beijing Institute of Radiation Medicine, Beijing, China; 2 College of Life Science, Hebei University, Baoding, Hebei 071002 China

**Keywords:** Magnetic fields, Neurobiological effects, Learning and memory, Emotional behavior, Anxiety, Depression

## Abstract

Magnetic fields are widely used in medical diagnostics because of their superior non-invasive properties. In addition, with the widespread use of magnetic fields in transportation and other areas, their potential hazards to human health and the assessment of their safety have attracted considerable attention. The effects of magnetic fields on living organisms have a long history. The biological effects of magnetic field exposure in mice and rats depend on the magnetic field strength, exposure time, and direction; depending on these and potentially other factors, magnetic fields can cause a series of neurobiological effects. We reviewed global research on the neurobiological effects of magnetic fields from recent years to provide an overview and insights into the underlying mechanisms. This review focuses on the biological effects of static and dynamic magnetic fields of different frequencies and intensities on animals and nerve cells and their mechanisms of action.

## Introduction

Magnetic fields (MF) transmit magnetic forces between physical objects. They have the radiation properties of particles and can be classified according to their strength as weak (< 1 mT), moderately strong (1 mT to 1 T), strong (1 T to 5 T), and ultra-strong (> 5 T) [1]. Magnetic fields can be divided into static magnetic fields (SMF) and dynamic magnetic fields (DMF), the latter includes alternating magnetic fields (AMF) and pulsating magnetic fields. An SMF, also known as a constant magnetic field, is a field whose strength and direction remain constant; in contrast, a DMF is an active directional field formed by convective alternations between one or several magnets [1, 2].

Currently, magnetic fields are widely used in medical and health care (such as magnetic resonance imaging and transcranial magnetic stimulation), transportation (magnetic levitation trains), national defense, and military fields. At the same time, magnetic fields pose potential hazards to human health, and their biological effects on the human body receive increasing attention. Studies have shown that the biological effects of magnetic fields are closely related to the frequency, intensity, exposure time, sex, age, general condition, and functional state of the affected organisms [3]. Magnetic field exposure affects the neurological [4], cardiovascular [2], endocrine [5], reproductive [6], immune [7], urinary [8], and muscular [9] systems. Epidemiological investigations and experimental studies have shown that the brain is the most sensitive target organ for magnetic field exposure [10], as evidenced by resulting cognitive impairment [11–15], abnormal emotional behavior [16–20], and electroencephalographic and histopathological changes in the brain.

Since magnetic fields have window effects [21, 22], exposure to magnetic fields at different frequencies, intensities, and times produces different biological effects. This review focuses on the biological effects of SMF and DMF of different frequencies and intensities on animals and nerve cells and their underlying mechanisms of action.

## Neurobiological effects of SMF

### Neurological effects of SMF on animals

Research on the biological effects of SMF has recently received increasing attention. Epidemiological investigations have shown that 8 T SMF exposure does not affect cognitive abilities such as short-term and working memory in humans [23, 24]. A 1.5-7 T magnetic resonance imaging scanner caused vertigo in the research staff [25].

Another study found that SMF exposure was closely related to learning, memory, and mood, and that different exposure intensities and times produced differential effects. Nakagawa et al. found that 0.6 T SMF exposure for 16 h/d over 4 d impaired learning and memory capacity in rats [11]. Another study exposed mice to a 2.0 T SMF for 100 min and found that spatial learning abilities significantly diminished after exposure [12]. Some studies have suggested that SMF exposure does not affect learning memory. Exposure to 0.6 T and 1.0 T SMFs did not affect volunteers’ visual and auditory working memory [26]. Hoyer et al. found that prolonged exposure to a 7 T SMF throughout the 17 d before birth (75 min/d) did not result in learning disabilities in adult mice [6]. High et al. found that a 9.4 T ultra-high SMF did not affect cognitive function in rats [27]. In general, the current conclusions about the effects of SMF on learning and memory are inconsistent.

Epidemiological investigations have shown that SMF exposure causes dementia and depression [28]. Long-term exposure to > 1.0 µT may increase the relative risk of suicide due to depression [29]. It has been shown that continuous exposure to 16 mT for 30 d reduces anxiety-like behaviors in male rats [2]. Exposure to a 5 mT SMF for 2 h/d over 5 d resulted in significant adverse effects on long-term anxiety-like behavior in mice [13], and repeated exposure to a 128 mT SMF for 5 d (1 h/d) resulted in anxiety-like behavior in rats [30]. Tang et al. also found that 50–200 mT SMF caused anxiety and depression-like behavior in rats [31]. Ultra-intense magnetic field studies have shown no deleterious effects in healthy or depressed mice exposed to 11.1–33.0 T SMF for 1 h or a 7 T SMF for 8 h. In contrast, these ultra-strong SMF have antidepressant potential [19]. It was further shown that ultra-high 23 T SMF exposure for 2 h enhanced the spatial memory capacity of mice and had anxiolytic effects [32]. These studies revealed that exposure to SMF with certain intensities leads to abnormal emotional behavior in animals.

In summary, SMF exposure can lead to alterations in learning memory and abnormal emotional behavior in animals, depending on the intensity of the exposure.

### Effects of SMF on nerve cells

The nervous system comprises two main cell types: neurons and glial cells. Glial cells are a class of non-neuronal cells that perform various functions, including providing structural support to neurons and insulating them to ensure more efficient information transfer between neurons. The biological effects of magnetic fields on nerve cells mainly promote cell differentiation and reduce the survival rate [33, 34].

#### Effects of SMF on neurons

Neurons are the most basic structural and functional units of the nervous system and are divided into two parts: the cell body and protrusions, including synapses. The cell body integrates incoming and transmits outgoing information. The synapse receives impulses from other neuronal axons and transmits them to the cell body. Studies have shown that the biological effects of SMF on neurons are related to field strength, but the findings are inconsistent. Ho et al. found that exposure to 0.5 T SMF induced proliferation and differentiation of neural progenitor cells in mice [38]. SMF exposure may affect normal neurogenesis by promoting mNPC differentiation and facilitating neuronal maturation and increasing neuron numbers [35]. In contrast, Valiron et al., investigating mouse hippocampal neurons exposed to 15 T for 1 h or 17 T for 1 h, found that both 15 and 17 T SMF caused significant neuronal damage and inhibited the formation of growth cones, resulting in sustained inhibition of the neuronal differentiation process [36]. These findings suggest that medium-strong SMF exposure may promote neuronal proliferation and differentiation, whereas ultra-high-intensity SMF exposure may inhibit the proliferation and growth of neurons.

#### Effects of SMF on glial cells

Neuroglia, referred to as glial cells, are a large class of cells in neural tissues with protrusions but no distinction between dendrites and axons and are widely distributed in the central and peripheral nervous system [37]. The ratio of glial cells to neurons is approximately 10:1 in mammals. Neuroglia in the central nervous system are mainly astrocytes, oligodendrocytes, and microglia. Astrocytes and oligodendrocytes are collectively known as macroglia. Few studies have investigated the effects of SMF on glial cells, and those mainly focused on glial cell proliferation and differentiation. 0.3T SMF (2 h/d, 14d) exposure promoted the differentiation of human oligodendrocyte precursor cells into myelinating mature oligodendrocytes, enhanced their myelinating ability, promoted the gene expression and secretion of neurotrophic factors BDNF and NT3, and additionally increased intracellular calcium influx and gene expression of CaV1.2 and CaV1.3 subunits. These findings suggest a positive effect of moderate intensity SMF exposure on glial cells [34]. Glioblastoma is the most common malignant astrocytoma in the central nervous system and is classified as malignant. Medeiros et al. studying the effects of different intensities of 0.1 T, 0.2 T, and 0.3 T SMF on human neuroblastoma cells, found that exposure to a 0.3 T SMF for 24 h resulted in a significant decrease in cell viability, and speculated that 0.3 T SMF have the potential for the treatment of neurological tumors [38]. da Costa et al. found that exposure of rat cortical astrocytes to 305 mT SMF impaired astrocyte oxidative homeostasis. In addition, SMF stimulation impaired cell viability by triggering cell cycle delay and death, and also altered mitochondrial function [39]. Evidently, magnetic fields with different strengths differentially affect glial cells.

Together, these results suggest that short-term exposure to SMF may lead to altered neuronal cell proliferation and differentiation, depending on the exposure dose.

## Neural effects of DMF

### Neurological effects of DMF on animals

Epidemiological studies have revealed that long-term exposure to extremely low-frequency magnetic fields (ELF-MFs) can cause a variety of neurological symptoms, such as vertigo, headache, dizziness, drowsiness, fatigue, insomnia, difficulty sleeping, dreaminess, irritability, forgetfulness, memory loss, and poor concentration [40, 41]. Global epidemiological investigations reported that ELF-MF exposure is closely associated with DNA damage [42, 43]. Many studies have indicated an association between long-term exposure to ELF-MFs and emotional behavior [44, 45]. Moreover, early studies have suggested that chronic ELF-MF exposure affects human cognitive function [46, 47]. Trimmel et al. revealed that a 50 Hz, 1 mT DMF reduces attention and memory abilities [48]. Corbacio et al. found that 60 Hz, 3 mT ELF-MF exposure may affect short-term learning abilities, which may be related to the brain’s plasticity [49]. However, one study found no effects of 20 and 400 µT, 40 Hz ELF-EMF exposure on cognitive functions in healthy men [50]. In addition, there is a significant reduction in the number of seizures in patients with epilepsy and an improvement in certain cognitive aspects in patients treated with repetitive transcranial magnetic stimulation (rTMS) [51, 52].

Several studies have reported the effects of AMF on learning memory. Mice exposed to magnetic radiation for 12 weeks, with a frequency of 50 Hz and an intensity of 1 mT, exhibited significantly decreased spatial learning ability [53]. Duan et al. exposed rats to 50 Hz, 8 mT ELF-MFs (4 h/day) for 4 weeks and observed decreased learning and memory capacities [54]. Furthermore, exposure to an AMF (0.5 Hz) with an induction value of 100 mT for 10 d reduced long-term reference memory in mice [55]. Fu et al. showed that continuous exposure to 50 Hz, 2 mT field strength for 25 d impaired spatial recognition memory in mice [56]. It has been suggested that long-term exposure to AMFs can impair learning and memory capacities, whereas some studies have shown that short-term exposure to AMFs also results in spatial learning and memory impairments. A Morris water maze study by Jadidi et al. found that exposure to a 50 Hz, 8 mT AMF for 20 min impaired the spatial memory capacity of rats [14].

Depending on AMF exposure time and field strength, different biological effects occur. It has been shown that 50 Hz, 1 mT ELF-MFs repeatedly applied for 28 d (2 h/d) enhanced learning and memory in cerebral ischemic rats [57]. Another study found that 40 Hz, 7 mT ELF-MF exposure for 4 weeks (15 min/d) significantly improved cognitive function in patients with ischemic stroke [58]. Akbarnejad et al. found that exposure to a 50 Hz, 10 mT magnetic fields for 14 d (60 min/d) improved the memory capacity of rats with Alzheimer’s disease [59]. Further studies have shown that long-term exposure to 50 Hz, 2 mT magnetic fields for 28 d (1 h/d and 4 h/d) enhanced spatial learning and memory in rats [60]. Vazquez et al. found that exposure to 60 Hz, 1 mT ELF-MFs for 9 d (2 h/d) enhanced memory capacity in adult rats [61]. ELF-MFs were found to improve visual memory and perceptual function in patients with Alzheimer’s disease [62]. Long-term exposure to AMFs may contribute to enhanced learning and memory capacities, whereas some studies have shown that short-term exposure to AMFs does not affect spatial learning and memory. A previous study reported that exposure to 50 Hz, 100 µT ELF-MFs for 12 weeks did not improve cognition and memory in rats with Alzheimer’s disease [63], and 50 Hz, 100 µT magnetic field exposures did not affect cognitive and memory functions in rats [64]. Fu et al. showed that continuous exposure to 25 Hz, 0.6 mT, and 50 Hz, 1.1 mT ELF-MFs for 7 d did not affect the spatial memory capacity of mice [56]. A study of non-spatial working memory with different intensities of a 50 Hz AMF found that 0.75, 7.5, and 75 µT field intensity exposure for 45 min did not affect the non-spatial memory capacity of mice [65]. Few studies have been conducted on the effects of pulsed magnetic fields on learning and memory. It was found that 10 mT, 20 Hz pulsed magnetic field exposure for 10 d (2 h/d) promoted learning and memory abilities in rats with streptozotocin-induced dementia, suggesting a potential role of pulsed magnetic fields in improving cognitive impairment [66]. Further studies are necessary to elucidate the role of AMFs in learning and memory dysfunctions.

In addition to the effects of DMF on cognitive function described in the previous paragraph, DMFs affect emotions. He et al. investigated the effects of ELF-MFs on the emotional behavior of rats subjected to a 50 Hz, 0.04 mT ELF-MF (4 h/d) for 4 weeks and showed that ELF-MFs induced significant increases in anxiety-like behavior [67]. Liu et al. exposed rats to magnetic field radiation of 2 mT for 4 h/d over 25 days. Following this exposure, the rats exhibited significantly increased anxiety-like behavior [68, 69]. Szemerszky et al. investigated magnetic field exposure in rats for 4 and 6 weeks and reported that 0.5 T ELF-MFs could significantly induce depressive behavior [70]. These data suggest that long-term exposure to ELF-MFs can cause an increase in anxiety- and depression-like behaviors.

However, some studies have shown decreased emotional behavior after magnetic field radiation. Kanno et al. exposed rats to rTMS at a frequency of 25 Hz. Three days after exposure, a significant improvement in anxiety-like behaviors was observed in the rats [71]. Sachdev et al. found that 1–25 Hz rTMS can improve depressive symptoms [72]. Meanwhile, continuous 15 Hz rTMS stimulation for 10 consecutive days decreased immobile time in forced swimming in rats, suggesting a therapeutic effect of rTMS on depression [73]. In addition, Lai et al. found that 50 Hz, 100 µT magnetic field radiation had no significant effect on the emotional behavior of rats [64]. Few studies have reported the effects of pulsed magnetic fields on emotional behavior. Wang et al. found that depressive behavior improved in rats after magnetic field exposure of 60 min/d for 14 days at 20 Hz and 1 mT [74]. Other studies have shown that depression-like behavior and cognitive dysfunction in chronically stressed rats are improved after long-term, low-dose rTMS at 20 Hz [75]. These studies further suggest the important role of rTMS in treating emotional abnormalities.

### Effects of DMFs on nerve cells

The biological effects of DMFs on neurogenic cells mainly focus on the cell proliferation, cell cycle, apoptosis [76], genotoxicity of cells [77], gene/protein expression [78], and neurogenesis [79]. The mechanisms lies in the fact that DMFs affects the concentration and balance of calcium ions, the surface molecules of cell membranes, and the production of ROS.

#### Effects of DMFs on neurons

Studies on neuronal responses to DMFs mainly focused on the effects of ELF-MFs on neurons. Su et al. found that exposure to 50 Hz, 2 mT 1 h, 6 h, and 24 h MFs during neuronal development did not significantly affect morphological parameters of rat cortical neurons [80]. Earlier studies had shown that 60 Hz, 2 mT MFs exposure did not increase γH2AX expression, DNA fragmentation and aneuploidy formation in mouse hippocampal neuronal cells HT22 cells [77]. However there were some studies that pointed to the in vitro neurotoxicity of ELF-MFs on a variety of nerve cells. Sul et al. found that 60 Hz, 2 mT MFs exposure at 1 h/d, 3 h/d, 6 h/d, for 7 and 14 days promoted the proliferation of cortical neuron HCN-2 cells, but did not affect cell cycle, morphological differentiation, or actin distribution [81]. Another study reported that 50 Hz, 1mT, 4 h/d, continuous ELF-MFs exposure for 1 d, 2 d, and 3 d promoted neuronal differentiation and neural synapse growth in embryonic neural stem cells by up-regulating the transient receptor potential specification and the expression of preneural genes (NeuroD and Ngn1) [82]. In addition, Höytö et al. found that 50 Hz, 100 µT MFs exposure induced an increase in mitochondrial activity as well as an increase in reactive oxygen species and lipid peroxidation levels in human SH-SY5Y neuroblastoma cells [83]. A study by Luo et al. found that exposure to 50 Hz MFs (1 mT or 3 mT for 24 h) affected intracellular calcium dynamics in neurons of the inner olfactory cortex through a calcium channel-independent mechanism [84]. de Groot et al. found that 50 Hz MFs exposure (1 mT for 7 d) affected depolarization and glutamate-induced increases in intracellular calcium concentration ([Ca2^+^]i) and neuronal synapse length, but not neuronal activity and cell viability [85]. This was confirmed by Calvo et al. who showed that 50 Hz AMF (1–15 mT) induced effects on neurons, and that Ca^2+^ ions were the cell membrane effectors of the interaction of 50 Hz AMFs with neuronal plasma membranes [86]. Raus et al. reported a significant decrease in the extent of neuronal damage and an increase in the responsiveness of hippocampal glial cells in ischemic gerbils caused by magnetic field radiation at 50 Hz with 0.5 mT [87]. Cuccurazzu found that 50 Hz, 1mT ELF-MFs (1–7 h/d, 7 d) exposure promoted neurogenesis of DG in mice by inducing expression of preneuronal genes (Mash1, Mash2, Hes1) and genes related to Cav1.2 channels [88]. Podda et al. investigated the effect of short-term exposure to an ELF-MF (50 Hz, 1 mT) for 6 days (3.5 h/d) on the survival of hippocampal neurons in mice and showed an increased survival rate of newborn hippocampal neurons [89]. There are few reports on the effects of pulsed magnetic fields on neurons. Li et al. reported that, after 7 d (20 min/d) exposure to a 110 mT pulsed magnetic field, offspring rats showed increased neurogranulin expression in hippocampal neurons [90]. These studies suggested that exposure to ELF-MFs might affect neurogenesis by altering Ca^2+^ signaling events, thereby resulting in neuronal dysfunction.

#### Effects of DMFs on glial cells

MFs have been applied to treat glioblastoma using heat therapy [20, 91, 92]. Rats and mice were exposed to AMFs (198 kHz) with intensities of 34–47 mT for 30 min, leading to gradually increasing glioblastoma cytotoxicity and cell mortality in their brains [93]. Zorzo et al. exposed rats to high-frequency rTMS with the following parameters: magnetic field frequency, 100 Hz; intensity, 330 mT; and exposure duration, 3 d; and reported a significant increase in neuronal activity in the posterior parietal cortex and hippocampal region of the rats; however, the treatment did not affect the density of rat astrocytes and microglia [94]. Exposure to 50 Hz ELF-MFs promoted cell proliferation in human neuroblastoma IMR32 by increasing the number of cell membrane voltage-gated Ca^2+^ channels and significantly inhibited apoptosis induced by puromycin or H_2_O_2_. It is suggested that increased Ca^2+^ inward flow at the cell membrane may be responsible for the magnetic field-induced regulation of neuronal cell proliferation and apoptosis [95]. Akbarnejad et al. reported that both 10 Hz, 50 G and 100 Hz, 100 G magnetic field exposure for 24 h enhanced the death of human glioblastoma cells and reduced their proliferative capacity [96]. These results indicate that magnetic field exposure may affect glial cell expression. Su et al. showed that exposure to a 50 Hz, 2 mT MFs did not cause DNA damage and abnormal cell function in neurogenic tumor cell lines (U251, A172, SH-SY5Y) and primary rat neurogenic cells (astrocytes, microglia) [80]. Another report showed that exposure to a 50 Hz, 100 µT MFs did not induce DNA damage and micronucleus formation in human SH-SY5Y neuroblastoma cells, but pre-exposure to a MFs altered cellular genotoxicity to menaquinone [97]. Koyama et al. reported that exposure of human glioma A172 cells to 60 Hz, 5 mT MFs alone did not affect the formation of apurinic/apyrimidinic (AP) sites in the genomic DNA of A172 cells, whereas the exposure could lead to an increase in the number of genotoxicant-induced AP sites in A172 cells, which possibly due to the fact that MFs exposure could enhance the activity or prolong the lifespan of free radical pairs [98]. Marcantonio et al. found that 50 Hz, 1 mT MFs exposure did not cause cell cycle changes or altered cell viability in neuroblastoma BE [2]C cells [99]. Sulpizio et al. reported that 50 Hz, 1 mT MFs exposure altered the proliferative state, cell growth pattern, and cytoskeletal organization of SH-SY5Y cells due to changes in the expression levels of proteins involved in cellular defense mechanisms, cellular organization, and biological behavior [100]. Benassi et al. showed that 50 Hz, 1 mT MFs exposure did not affect the proliferation, differentiation, shape and morphology of SH-SY5Y cells, but impaired redox homeostasis and triggered an increase in protein carbonylation [101]. Besides that, it was found that 50 Hz (100 µT, 42 h intermittent exposure, 3 h on/3 h off) ELF-MFs exposure promoted human neuroblastoma NB69 cell proliferation [102], while 60 Hz (0.3–1.2 G, 3–72 h) MFs exposure also enhanced the proliferation of astrocytoma 132-1N1 cells, in which protein kinase C may play an important role [103].

In summary, whether different magnetic field exposures induce alterations in cognition, emotion, and nerve cells and whether these effects are beneficial or detrimental has not been clarified conclusively. The neurocellular effect of MFs are determined by the physical parameters of the MFs and cellular properties. The inconsistent reports were closely related to the magnetic field frequency, intensity, and duration of exposure (Tables 1, 2, 3 and 4).

**Table 1:** Summary of SMF bioeffects on neurobiological behavior including learning memory and emotional behavior in animals

Neurobehavior	Effect direction	Authors	Rodent models	Effective exposure conditions	Results
Learning and memory	Detrimental	Nakagawa et al. [11]	Rats	0.6 T; 16 h/d; 4 d	Impairment of learning and memory capacity
		Saeedi et al. [13]	Mice	5 mT; 2 h/d; 5 d	Significant adverse effects on memory
		Levine et al. [12]	Mice	2 T; 100 min	Significantly weakened spatial learning ability
		Ammari et al. [30]	Rats	128 mT; 1 h/d; 5 d	Impairment of spatial learning ability
	Beneficial	Khan et al. [32]	Mice	23 T; 2 h	Enhancement of spatial memory ability
	No effect	Hoyer et al. [6]	Mice	7 T; 75 min/d; 17 d	No learning disabilities occurred
Emotional Behavior	Detrimental	Saeedi et al. [13]	Mice	5 mT; 2 h/d; 5 d	Developed chronic anxiety-like behavior
		Tang et al. [31]	Rats	50 mT; 100 mT; 200 mT; 15d	Caused anxiety and depression
	Beneficial	Tasic et al. [2]	Rats	16 mT; 30 d	Reduced anxiety-like behavior
		Khan et al. [32]	Mice	23 T; 2 h	Anti-anxiety effect
	No effect	High et al. [27]	Rats	9.4 T	No effect on cognitive function
		Lv et al. [19]	Mice	11.1–33.0 T; 1 h	No harmful effects
		Lv et al. [19]	Mice	7 T; 8 h	No harmful effects

**Table 2:** Summary of DMF bioeffects on neurobiological behavior including learning memory and emotional behavior in animals

Neurobehavior	Effect direction	Authors	Rodent models	Effective exposure conditions	Results
Learning and memory	Detrimental	Cui et al. [53]	Mice	50 Hz; 1 mT; 3 months	Impairment of space learning functions
		Duan et al. [54]	Rats	50 Hz; 8 mT; 4 h/d; 30 d	Damage to learning and memory skills
		Li et al. [55]	Mice	0.5 Hz; 1 T; 10 d	Compromised access to long-term reference memory
		Fu et al. [56]	Mice	50 Hz; 2 mT; 25 d	Impairment of spatial recognition memory
		Jadidi et al. [14]	Rats	50 Hz; 8 mT; 20 min	Negative effects on spatial memory ability
	Beneficial	Gao et al. [57]	Cerebral ischemia in rats	50 Hz; 1 mT; 2 h/d; 28 d	Enhancement of learning and memory
		Akbamejad et al. [59]	Alzheimer’s disease rats	50 Hz; 10 mT; 60 min/d; 14 d	Improved memory
		Liu et al. [60]	Rats	50 Hz; 2 mT; 1 h/d for 28 d; 4 h/d; 28 d	Enhancement of spatial learning and memory
		Vazquez-Garcia et al. [61]	Rats	60 Hz; 1 mT; 2 h/d; 9 d	Enhanced social recognition memory
		Li et al. [66]	Demented rats	10 mT; 20 Hz; 2 h/d; 10 d	Promoted learning and memory
	No effect	Zhang et al. [63]	Alzheimer’s rats	50 Hz; 100 µT; 3 months	No improvements in cognition and memory
		Lai et al. [64]	Rats	50 Hz; 100 µT	No significant effect
		Fu et al. [56]	Mice	25 Hz; 0.6 mT; 50 Hz; 1.1 mT; 7 d	No effect on spatial memory abilities
		Sienkiewicz et al. [65]	Mice	50 Hz; 0.75 µT; 7.5 µT; 75 µT; 45 min	No effect on non-spatial memory abilities
Emotional Behavior	Detrimental	He et al. [67]	Rats	50 Hz; 2 mT; 4 h/d; 4 weeks	Caused anxiety
		Liu et al. [68, 69]	Rats	50 Hz; 2 mT; 4 h/d; 25 d	Increased anxiety-like behavior
	Beneficial	Kanno et al. [71]	Rats	25 Hz; 3 d	Anxiolytic effect
		Sachdev et al. [72]	Rats; Mice	1–25 Hz	Significant antidepressant effects
		Tsutsumi et al. [73]	Rats	15 Hz; 10 d	Therapeutic effects on depression
		Wang et al. [74]	Rats	20 Hz; 1 mT; 60 min/d; 14 d	Antidepressant effect
		Yang et al. [75]	Chronic Stressed Rats	20 Hz; 1 mT; 60 min/d; 14 d	Improved depression-like behavior and cognitive dysfunction
	No effect	Lai et al. [64]	Rats	50 Hz; 100 µT; 24 weeks	No significant effect

**Table 3:** Summary of MF bioeffects on neurogenic cells

Type of MFs	Authors	Neurogenic cell models	Effective exposure conditions	Results
SMF	Ho et al. [35]	Mouse neural progenitor cells	0.5T; 24 h/; 7d	Promoted differentiation
	Prasad et al. [34]	Human oligodendrocyte precursor cells	0.3T; 2 h/d; 2weeks	Stimulated differentiation
	Valiron et al. [36]	Mouse hippocampal neuron	15T; 17T; 1 h	Injured neuron
	Medeiros et al. [38]	Human neuroblastoma cells	0.1T; 0.2T; 0.3T; 24 h	Decreased cell viability
	da Costa et al. [39]	Rat cortical astrocytes	305mT; 5,15,30,40 min/d; 7d	Decreased antioxidant capacity
DMF	Luo et al. [84]	Neuron	50 Hz; 1,3mT; 24 h	Affected intracellular calcium dynamics
	de Groot et al. [85]	Neuron	50 Hz; 1mT; 7d	Increased Ca^2+^ inward flow
	Calvo et al. [86]	Neuron	50 Hz; 1-15mT	Increased Ca^2+^ inward flow
	Raus et al. [87]	Neuron	50 Hz; 0.5mT	Reliefed neuronal damage
	Cuccurazzu et al. [88]	Neuron	50 Hz; 1mT; 1–7 h/d, 7d	Promoted neurogenesis
	Podda et al. [89]	Neuron	50 Hz; 1mT; 3.5 h/d, 6d	Promoted neuronal survival
	Li et al. [90]	Neuron	110mT; 20 min/d, 7d	Promoted neuronal maturation
	Sul et al. [81]	Cortical neuron HCN-2 cells	60 Hz; 2mT; 1,3,6 h/d; 14d	Promoted proliferation
	Ma et al. [82]	Embryonic neural stem cells	50 Hz; 1mT; 4 h/d; 1d,2d,3d	Promoted differentiation
	Höytö et al. [83]	Human SH-SY5Y neuroblastoma cells	50 Hz; 100 µT	Increased mitochondrial activity
	Grassi et al. [95]	Human neuroblastoma IMR32 cells	50 Hz; 1mT; 24,48,72 h	Enhanced proliferation
	Trillo et al. [102]	Human neuroblastoma NB69 cells	50 Hz; 100µT, 42 h intermittent exposure, 3 h on/3 h off	Promoted proliferation
	Wel et al. [103]	Astrocytoma 132-1N1 cells	60 Hz; 0.3–1.2 G; 3–72 h	Enhanced proliferation
	Koyama et al. [98]	Human glioma A172 cells	60 Hz; 5mT	Enhanced the activity of free radical pairs
	Akbarnejad et al. [96]	Human glioblastoma cells	10 Hz, 50 G; 100 Hz, 100 G; 24 h	Enhanced cell death
	Sulpizio et al. [100]	SH-SY5Y cells	50 Hz; 1mT	Promoted proliferation
	Benassi et al. [101]	SH-SY5Y cells	50 Hz; 1mT	Impaired the oxidation state
	Su et al. [80]	Neurogenic tumor cells, Primary cultured rat neurogenic cells	50 Hz; 2mT	No significant effect
	Luukkonen et al. [97]	Human SH-SY5Y neuroblastoma cells	50 Hz; 100µT	No significant effect
	Marcantonio et al. [99]	Neuroblastoma BE [2]C cell	50 Hz; 1mT	No significant effect
	Su et al. [80]	Neuron	50 Hz; 2mT; 1,6,24 h	No significant effect

**Table 4:** Percentage of studies on MFs exposure on learning memory and emotional behavior in animals with certain effect directions

Type of magnetic field	Type of research	Effect direction		
		Beneficial	Detrimental	No effect
Static magnetic fields	Effects on learning and memory in animals	12.5%	62.5%	25%
	Effects on the emotional behavior of animals	50%	40%	10%
Dynamic magnetic fields	Effects on learning and memory in animals	26%	44%	30%
	Effects on the emotional behavior of animals	46%	46%	8%

## Effects of MFs on neurotransmitters

Neurotransmitters are chemicals that transmit information between neurons or between neurons and effector cells. Four categories of neurotransmitters are based on their chemical characteristics: choline, such as acetylcholine (Ach), monoamines, such as norepinephrine, dopamine (DA), and 5-hydroxytryptamine, amino acids, such as glutamate and gamma amino butyric acid, and neuropeptides.

Several studies have reported the effects of SMF on neurotransmitters. Cheng et al. exposed rats to a 0.5 T or 1 T SMF and found no significant changes in neurotransmitter levels, including choline, γ-aminobutyric acid, and DA [104]. Furthermore, exposure to an SMF of 128 mT for 1 h/d over 5 d led to increased norepinephrine levels in the gastrocnemius muscle [105]. These studies suggest that a certain intensity of SMF radiation can alter the metabolism of neurotransmitters.

The effects of AMF on neurotransmitters have also been previously investigated. Electromagnetic fields could reduce the affinity constant of 5-HT and its receptor, thus inhibiting the activity of 5-HT [106]. The blockade of signaling pathways by inhibiting the binding of receptors to ligands might be a mechanism of action of MFs. Lai et al. (133) found that 60 Hz, 2 mT MFs could reduce cholinergic activity by blocking the uptake of acetylcholine in anterior ventricular cortex and hippocampus of rat. It was believed that MFs could induce analgesic effects through opiate receptors. The effect of MFs on opiate receptors was confirmed, suggesting that MFs not only reduce receptor function, but also up-regulate receptor expression. Sieroń et al. exposed rats to an ELF-MF with a frequency of 10 Hz and intensities of 1.8–3.8 mT for 1 h/d over 14 d and found that DA and 5-HT turnover rates increased significantly in the rat frontal cortex [107]. In addition, Janac et al. revealed that exposure to an AMF (50 Hz, 0.5 mT) for 7 d decreased the affinity of serotonin 5-HT_2A_ receptors and increased their density in the prefrontal cortex of rats [108]. Sieroń et al. exposed rats to an AMF with a frequency of 10 Hz and intensities of 1.8–3.8 mT for 14 consecutive days, and reported that the central dopamine D1 receptor responsiveness in the rats decreased [109]. Further, exposure to an AMF (20 Hz, 14 mT ELF-MF) decreased the DR2 density in rat dorsal hippocampal neurons, and exposure to a 60 Hz, 2.4 mT ELF-MF for 14 d resulted in enhanced dopamine DR1/DR2 in the rat striatal complex. Exposure to a 50 Hz, 0.5 mT ELF-MF for 7 d affected cortical serotonergic neurotransmission in rats in a time-dependent manner, but no obvious changes in DR1 and DR2 were observed [108]. Moreover, some studies have shown that magnetic fields may exert antidepressant effects by altering levels of amino acid-like neurotransmitters in the brain [110, 111]. Together, these results suggest that exposure to MFs may lead to abnormal neurotransmitter levels in the brain, depending on the radiation dose.

## Mechanisms of action of MFs

Current research on mechanism of the biological effects of MFs mainly focuses on effects of MFs on cell membranes, membrane receptors and signaling pathways, effects of MFs on cell proliferation and apoptosis and their mechanisms, as well as chromosomal aberrations. It is believed that the cell membrane is the first target of MFs on cells, and the cell membrane plays a major role in the organism’s response to MFs. Santoro et al. found that 50 Hz, 2 mT MFs exposure not only induced a decrease in cytosolic flow rate, reorganization of cytoskeletal components, and loss of microvilli, but also interfered with protein phosphorylatioy [112]. It could be hypothesized that MFs might interfere with the initiation of cellular signaling by altering the structure of membrane protein receptors. Another experiment showed that low-intensity pulsed electromagnetic fields caused a large increase in the number of electrons on both sides of the cell membrane [113], activated the cell membrane action potential, potassium channels and increased the activity of Na^+^-K^+^-ATPase and Ca^2+^-Mg^2+^-ATPase, which resulted in a significant reduction in the fluidity of rat hippocampal neuron cell membranes. On the other hand, ELF-MFs, while affecting the basic structure of the cell membrane, in a study of enzyme-loaded monolayers of liposomes exposed to 7-Hz, 50-µT MFs for 60 min, the internal liposomal enzyme content was showed to have an elevated concentration. It was theorized that MFs enhanced permeability of the cell membrane [114], thereby regulating the intra- and extracellular osmotic pressure.

Furthermore, MFs have an effect on cell membrane potential. It had been reported that 50 Hz, 0.5 mT MFs could change voltage-gated K^+^ channel properties, which significantly inhibited the magnitude of potassium channel currents in neuron cell membranes, and that the changes in the K^+^ channel properties in turn have an effect on the depolarization and hyperpolarization processes which generate action potentials [115]. Li Gang et al. found that 50 Hz, 1 mT industrial frequency MF inhibited voltage-dependent K^+^ channel currents in neurons [116].In contrast, Shen et al. experimentally showed that an industrial frequency magnetic field with 125 mT had almost no effect on the current generated by K^+^ ion channels [117]. In a study of neuronal cells of the inner olfactory cortex, it was found that 50 Hz, 1 mT MFs exposure for 24 h had no effect on calcium channels in the cell membranes, whereas it reducd the maximum amplitude of the calcium voltage induced by high potassium [84]. In a study of Na^+^ channels, neurons were exposed to 30 mT SMF, and it was found that SMF shifted the Na^+^ channel activation potential of the neuronal cells in the direction of hyperpolarization, and the peak value of Na^+^ current increased [118]. Consequently, the action of MFs on cell membrane potential is through the effect on Na^+^, K^+^ and Ca^2+^,, although the effect varied depending on the physical parameters of MFs, more studies showed that MFs could increase the inward flow of Na^+^ and Ca^2+^, induce the depolarization of the cell membrane, and inhibit the K^+^ channel.

**Fig. 1:**
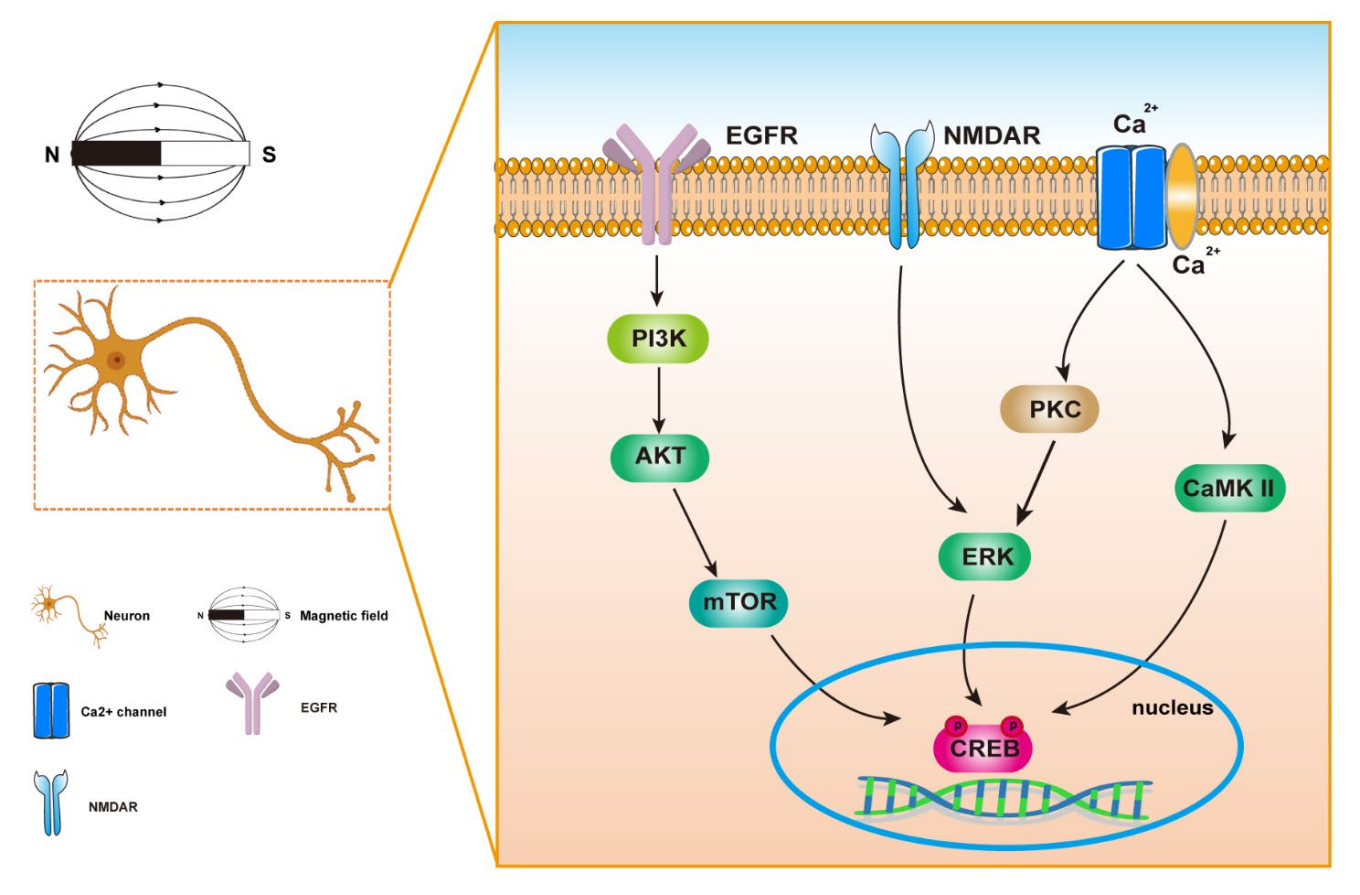
Effects of MF on neuronal signaling pathway. MF mainly affects EGFR-Akt-CREB, NMDAR-ERK-CREB, Ca^2+^ -dependent CaMK II/CREB signaling pathways

In addition, the cell membrane receptors and signaling pathways are also the targets of action of MFs. Duan et al. found that 50 Hz ELF-MFs (8 mT, 28 d) exposure significantly increased glutamate and aspartate receptors and intracellular calcium concentration in hippocampal cells, and decreased phosphorylation of extracellular regulated signal kinase ERK and binding protein CREB [54]. Park et al. found that enhanced neuronal differentiation induced by exposure to ELF-MFs was associated with phosphorylation of CREB, which could be traced upstream to the activation of epidermal growth factor receptor (EGFR) through Akt signaling [119]. Ozgun et al. found that exposure to 50 Hz, 1 mT ELF-MFs increased neuronal markers, c-Fos, and synapse growth. These effects could be completely reversed by NMDA receptor antagonists, showing severe NMDA receptor-dependent [120]. It was suggested that effects of ELF-MFs exposure on neuronal differentiation aroused from effects on NMDA receptors, and that NMDA receptor activation leaded to Ca2^+^ inward flow and Ca^2+^-dependent cascade responses. This was also confirmed by Manikonda et al., who found that ELF-MFs could cause malfunctioning NMDA receptor activity by altering Ca^2+^ homeostasis [121]. Consequently, it is hypothesized that ELF-MFs exposure may activate NMDA receptors and Ca^2+^-dependent pathways, which in turn activates the EGFR-Akt-CREB cascade. In addition, it was found that 50 Hz, 8mT ELF-MFs (4 h/d for 28d) increased Gi protein, IP3, DAG, Ca^2+^, PKA, PKC β, Ca- and PP2B, however, CaMK II, PKC α, and BDNF were decreased by ELF-MFs [122]. These results suggest that ELF-MFs may mediate calcium signaling and dual-messengers through Ca^2+^/ CaMKII/CREB/BDNF and DG/PKC/MAPK signaling pathways.(Fig. 1).

## Conclusion

In summary, this article reviews the progress in research on the neural effects of SMF and DMF at the level of animal models and nerve cells in recent years. The number of studies on the effects of MFs on learning memory, emotional behavior, nerve cells, and neurotransmitters is gradually increasing, but owing to the diversity of MF parameters, experimental subjects, and conditions, the conclusions are inconsistent. Certain conditions of MF exposure can lead to changes in emotional behavior and learning memory and cause or relieve anxiety-like and depressive behaviors, with or without significant effects. The biological effects of MFs on neurons and glial cells include alterations in cell proliferation, cell cycle distribution, and apoptosis. However, some problems remain unclear. Due to the unspecific nature of MFs, their neurobiological effects are difficult to target experimentally. Urgent problems to be solved by future research include how to establish proper experimental animal and neural cell models, and how to select the appropriate MF exposure intensity and time.

## Data Availability

No datasets were generated or analysed during the current study.

## Abbreviations

5-HIAA: 5-hydroxyindoleacetic acid
5-HT: 5-hydroxytryptamine
Ach: Acetylcholine
AMF: Alternating magnetic fields
DA: Dopamine
DMF: Dynamic magnetic fields
ELF-MFs: Extremely low-frequency magnetic fields
MF: Magnetic fields
rTMS: Repetitive transcranial magnetic stimulation
SMF: Static magnetic fields
ERK: Extracellular signal-regulated kinase
CREB: Cyclic-AMP response element binding
EGFR: Epidermal growth factor receptor
MAPK: Mitogen-activated protein kinase
NMDA: N-methyl-D-aspartate
IP3: Inositol triphosphate
BDNF: Brain-derived neurotrophic factor
DAG: Diacylglycerol
PKA: Protein kinase A
PKCβ: Protein kinase Cβ
PKCα: Protein kinase Cα
PP2B: Protein phosphatase 2B
